# Extracting complexity waveforms from one-dimensional signals

**DOI:** 10.1186/1753-4631-3-8

**Published:** 2009-08-14

**Authors:** Aleksandar Kalauzi, Tijana Bojić, Ljubisav Rakić

**Affiliations:** 1 Department for Life Sciences, Institute for Multidisciplinary Research, University of Belgrade Kneza Višeslava 1, 11000 Belgrade Serbia; 2 Institute for Pharmacology, Clinical Pharmacology and Toxicology, School of Medicine, University of Belgrade Dr Subotića 1, 11000 Belgrade Serbia; 3 Serbian Academy of Sciences and Arts, Belgrade, Knez Mihailova 35, 11000 Belgrade Serbia

## Abstract

**Background:**

Nonlinear methods provide a direct way of estimating complexity of one-dimensional sampled signals through calculation of Higuchi's fractal dimension (1<*FD<2*). In most cases the signal is treated as being characterized by one value of *FD *and consequently analyzed as one epoch or, if divided into more epochs, often only mean and standard deviation of epoch *FD *are calculated. If its complexity variation (or running fractal dimension), *FD(t)*, is to be extracted, a moving window (epoch) approach is needed. However, due to low-pass filtering properties of moving windows, short epochs are preferred. Since Higuchi's method is based on consecutive reduction of signal sampling frequency, it is not suitable for estimating *FD *of very short epochs (*N *< 100 samples).

**Results:**

In this work we propose a new and simple way to estimate *FD *for *N *< 100 by introducing 'normalized length density' of a signal epoch,

where *y*_*n*_(*i*) represents the *i*th signal sample after amplitude normalization. The actual calculation of signal *FD *is based on construction of a monotonic calibration curve, *FD *= f(*NLD*), on a set of Weierstrass functions, for which *FD *values are given theoretically. The two existing methods, Higuchi's and consecutive differences, applied simultaneously on signals with constant *FD *(white noise and Brownian motion), showed that standard deviation of calculated window *FD *(*FD*_*w*_) increased sharply as the epoch became shorter. However, in case of the new NLD method a considerably lower scattering was obtained, especially for *N *< 30, at the expense of some lower accuracy in calculating average *FD*_*w*_. Consequently, more accurate reconstruction of *FD *waveforms was obtained when synthetic signals were analyzed, containig short alternating epochs of two or three different *FD *values. Additionally, scatter plots of *FD*_*w *_of an occipital human EEG signal for 10 sample epochs demontrated that Higuchi's estimations for some epochs exceeded the theoretical *FD *limits, while NLD-derived values did not.

**Conclusion:**

The presented approach was more accurate than the existing two methods in *FD*(*t*) extraction for very short epochs and could be used in physiological signals when *FD *is expected to change abruptly, such as short phasic phenomena or transient artefacts, as well as in other fields of science.

## Background

**Figure 1:**
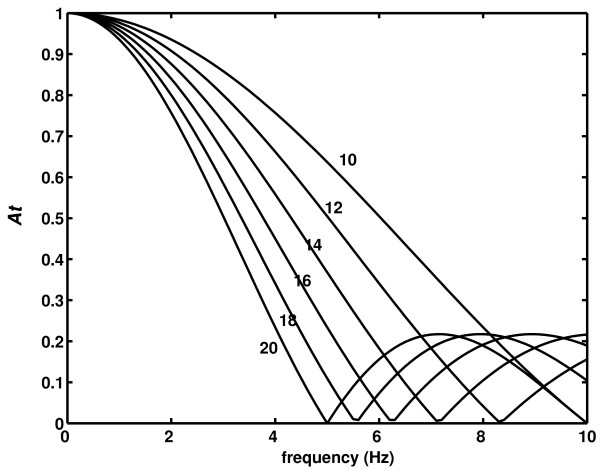
**Low-pass filtering properties of a series of moving windows**. *A*_*t *_– amplitude attenuation; different lines correspond to different window lengths (*N*_*w *_= 10,12,...,20 samples); signal sampled at *f*_*s *_= 100 samples/s.

It is well known that only in mathematically generated one-dimensional signals one can expect a particular quantity *Q*, characterizing the signal, to be constant (independent of the domain variable, usually time). In other words, in real (and not only biomedical) signals, *Q *= *Q*(*t*), and in many cases it is of interest to calculate such a waveform as accurately as possible. Along that line, the usual approach is to introduce a moving window, calculate the quantity *Q *for this subset of samples, *Q*_*w*_, and extract an approximation of *Q*(*t*) in the form of *Q*_*w*_(*t*_*i*_), where *t*_*i *_= *t*_*o*_+(*i*-1)*t*_*s *_denotes the *i*th window position along the signal, *t*_*o *_and *t*_*s *_being the initial position and moving window step, respectively. In case of amplitudes, as it is known from the classical Fourier signal analysis, a moving window acts as a low-pass filter – attenuating amplitudes of signal oscillations according to the equation

(1)

where *At*(*f*_*n*_) denotes amplitude attenuation at frequency *f*_*n*_; *f*_*n *_- *n*th Fourier frequency; *L*_*w *_window length (Fig. 1). The first cutoff frequency, *f*_*c*_, is then calculated as

(2)

where *f*_*s *_represents the sampling frequency, *N*_*w *_number of samples in the window.

According to equation (2), in order to extract accurately the waveform *Q*(*t*), *i.e*. avoid the attenuation of as many Fourier components as possible, it is desirable to have large values of *f*_*c*_, according to (2) small values of *N*_*w *_(short windows) for a given sampling frequency. Importance and limitations of estimating nonlinear properties of biological (particularly EEG) signals for short epochs has already been recognized (cf. [1,2]).

Although these filtering properties were derived for oscillations (waveforms) of signal amplitudes, we showed in our previous work dealing with meteorological data (cf. [3,4]) that they are also valid for oscillations of signal complexity, expressed quantitatively as "running fractal dimension" *Q*(*t*) = *FD*(*t*) (cf. [5]).

Estimation of fractal dimension of one-dimensional sampled signals is usually performed by Higuchi's algorithm (cf. [6]), although other methods have also been proposed (cf. [7,8]) and their performance evaluated (cf. [9]). In Higuchi's method, the signal is observed as consisting of a time sequence *x*(1), *x*(2),..., *x*(*N*) and *k *new self-similar time series are constructed as:



for *m *= 1, 2,..., *k*; where *m *is the initial time; *k *= 2,..., *k*_*max*_, being the degree of time stretch, int(*r*) is integer part of a real number *r*. The length *L*_*m*_(*k*) is computed for each of the *k *time series or curves as

(3)

*L*_*m*_(*k*) is then averaged for all *m*, forming the mean value of the curve length *L(k)*, for each *k = *2,...,*k*_*max *_as

(4)

An array of mean values *L*(*k*) is thus obtained and the *FD *estimated as the slope of least squares linear best fit from the plot of ln(*L*(*k*)) versus ln(1/*k*). However, since Higuchi's method is based on consecutive reduction of signal sampling frequency, it is not suitable for estimating *FD *of very short epochs (*N *< 30 samples), while 100 samples could be regarded as a conventional low limit for its application (cf. [5]). Another disadvantage of this method is the fact that the maximal degree of reduction (*k*_*max*_) is left to be determined arbitrarily by the researcher (cf. [10]). In our previous paper (cf. [8]), we described an original method for calculating *FD*, using consecutive differences (*CD*) of one-dimensional sampled signals. Namely, if we denote mean absolute values of the *n*th order consecutive finite differences of a signal *y(t) *with *m*^(*n*)^_*y*_, we found that logarithms of *m*^(*n*)^_*y*_, *n *= 2, 3,..., *n*_*max*_, were linearly dependent on *n*:

(5)

with stable slopes and *Y-*intercepts proportional to signal *FD*. To establish a relation between *Y*_*int *_and signal fractal dimension, we used a family of Weierstrass functions, which have a theoretically defined value of fractal dimension. Since we found that their *FD *values were linearly dependent on *Y*_*int*_

(6)

we were able to calculate parameters *A*(*n*_*max*_) and *B*(*n*_*max*_) for *n*_*max *_= 3,...,7. In this method, the need to choose a value for *k*_*max *_is eliminated. More, introducing *n*_*max *_instead of *k*_*max *_did not mean substituting one indeterminacy with another, since the smallest numerical error, on the used set of Weierstrass function, was obtained with *n*_*max *_= 3.

## Results

### Normalized length density

How to approach measuring of signal complexity on very short epochs? One of the ways could be to count the local extrema, where the signal complexity is expected to be directly proportional to their number. However, complexity is not equal if there are e.g. 3 local extrema on 5 or 50 samples. Therefore, if *N*_*le *_denotes the number of local extrema and *N *number of samples, one may introduce 'local extrema density', *L*_*ed*_, as the measure of complexity:

(7)

**Figure 2:**
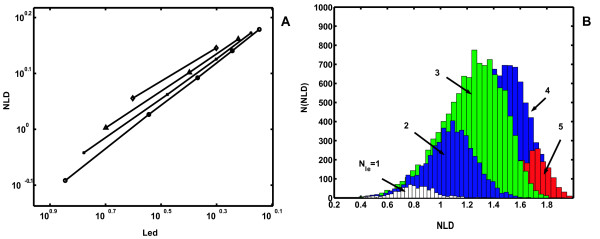
**A. Linear dependence of normalized length density (*NLD*) on the local extrema density (*Led*) in the log-log scale**. Data on each line were derived from 30000 short epochs with different lengths (o - 7; x - 6; Δ - 5; ◇ - 4 samples). Epochs consisted of randomly generated samples in the range (0,1). **B**. Distributions of number of generated epochs, N(*NLD*), by their *NLD *values, derived from the 30000 seven-sample epochs from panel **A**. Histograms differ in the number of local extrema detected on the epochs (*N*_*le*_), and positions of their peaks correspond to ordinate values of circles on **A**.

But this quantity has a substantial drawback: because both *N*_*le *_and *N *are integers, *L*_*ed *_tends to group such short signal epochs into 'quantized' classes. For example, if *N *= 4, the method is able to classify all epochs into only three classes of complexity, having *L*_*ed *_= 0.00; 0.25; 0.50 (values of 0.75 and 1.00 should be excluded since we do not know whether edge samples are local extrema or not). Obviously, a continuous measure of signal complexity would be more suitable. We propose that such a quantity could be, 'normalized length density' (*NLD*). In fact, it is the signal length divided by the number of samples, and normalized for average signal amplitude and it is proportional (Fig. 2A) to the local extrema density:

(8)

where *y*_*n*_(*i*) represents *i*th signal sample after amplitude normalization:

(9)

where

(10)

**Figure 3:**
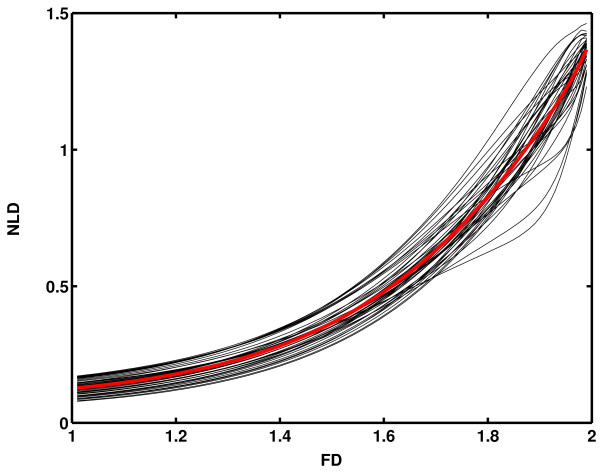
**Relationship between theoretical fractal dimension (*FD*) of 3960 tested Weierstrass functions and their normalized length density (*NLD*)**. Forty thin black lines correspond to Weierstrass functions with forty fixed values of the intrinsic parameter *γ*, thick red line to their average.

How to relate *NLD *with *FD*? For this purpose we used the same set of Weierstrass functions as in [8]:

(11)

(γ > 1, 0<H<1), where *FD *= 2-*H *is given theoretically. A family of these functions was generated, with parameter values being *γ *= 1.1,1.2,...,5.0 (*N*_*γ *_= 40), *H *= 0.99,0.98,...,0.01 (*N*_*H *_= 99). The latter values resulted in an even distribution of *FD *values across their possible numerical range (1 <*FD *< 2). The whole set, therefore, numbered *N*_*γ*_*N*_*H *_= 3960 functions. Sampling frequency and signal duration were chosen to resemble a biological signal: *f*_*s *_= 256 samp/s, *T *= 30s, making the total number of samples within each Weierstrass function to be *N *= 7680. For each set of 99 Weierstrass functions, having a fixed value of *γ*, *NLD*_*γ *_= *φ*_*γ*_(*FD*) functions were calculated after amplitude normalization according to formulas (9) and (10) and presented as 40 thin black lines on Fig. 3. Since real biological signals are not characterized by any intrinsic parameter such as *γ*, by averaging all 40 *NLD*_*γ *_values for a fixed *FD*, inverse of the final calibration curve

(12)

was calculated and presented as a thick red line on Fig. 3. As this and its inverse function *FD *= φ^-1^(*NLD*) = f(*NLD*) turned to be monotonous, the latter fulfilled the necessary condition to be used as a calibration curve in further calculations.

For computational purposes, a mathematical model of this relation must be established. In this work we tested two models:

- logarithmic model: *FD *= *a *log (*NLD *- *NLD*_*o*_) + *C*

- power model: *FD *= *a *(*NLD *- *NLD*_*o*_)^*k*^.

**Figure 4:**
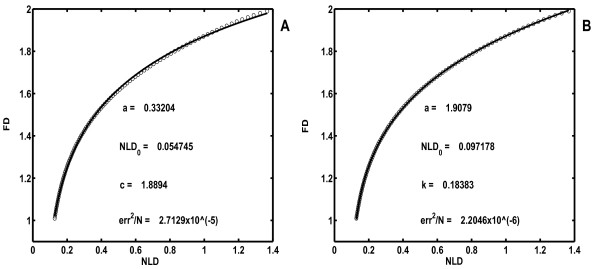
**Circles: fractal dimension (*FD*), as a function of normalized length density (NLD), obtained from a set of Weierstrass functions (*FD *= 1.01 – 1.99; *γ *= 1.1 – 5.0)**. Solid line: nonlinear fitting with two mathematical models – logarithmic (**A**) and power (**B**).

Nonlinear fitting was performed on the obtained points *FD *= f(*NLD*) (circles, Fig. 4) for both mathematical models. As can be seen, the power model showed better results (smaller square fitting error per point). A better matching of the power model to the experimental points could also be seen visually, especially for higher values of *NLD *and *FD*.

When extracting complexity waveforms from one-dimensional signals, a problem arises which is not present when conventional *FD *measurements are applied (averaging of window *FD *values for the whole signal). Namely, since in every natural signal both amplitude and complexity simultaneously vary, it is essential to eliminate, as much as possible, influence of amplitude variations on complexity measurements. Fortunately, Higuchi's method is invariant to amplitude variations. However, this is not the case when NLD analysis is performed. Two procedures are possible for signal amplitude normalization: a) to normalize signal amplitudes for the whole signal, by applying formulas (9) and (10) before the NLD procedure. Such a procedure is in accordance with the way the calibration curves, presented on Fig. 3 and Fig. 4B were obtained after amplitude normalization of the Weierstrass functions. However, this version (integral normalization, IN) is not entirely immune on signal amplitude variations that necessarily occur while the window of analysis moves along the signal. b) According to the other version of the method, amplitude normalization is to be performed on every part of the analyzed signal selected by the moving window (window normalization, WN). Each of these two versions of the NLD method showed more or less accurate results in extracting complexity waveforms for very short signal epochs, depending on the measurement conditions. Therefore, the problem of current signal amplitude variations and elimination of their influence on *FD*(*t*) is still to be elucidated in future studies.

### Dependence of accuracy of *FD *calculation on epoch length

**Figure 5:**
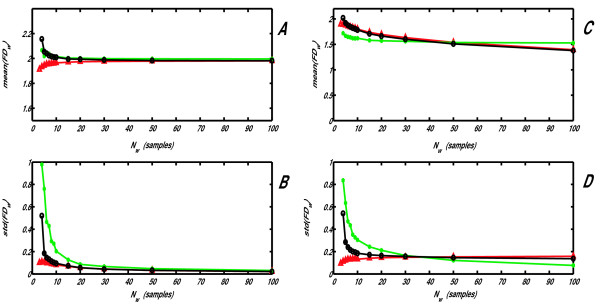
Mean (A, C) and standard deviation (B, D) of window fractal dimension (*FD*_*w*_) values, obtained by analyzing white noise (A, B) and Brownian motion (C, D) with Higuchi's (green, *), consecutive differences (black, o) and the power model version (WN) of the NLD method (red, Δ).

All natural signals are of variable complexity. Accuracy of any method for measuring *FD *could be estimated by scattering (standard deviation) of epoch (window) *FD *values, *FD*_*w*_, if signals with constant *FD *are being analyzed (such as white noise, *FD *= 2, and fractal Brownian motion, *FD *= 1.5). Standard deviation of all measured *FD*_*w *_increases with shortening of windows, causing numerical errors. We compared this scattering obtained by Higuchi's, consecutive differences and NLD method (power model version, WN) on these two types of noise (Fig. 5). Integral normalization was also performed, but since the results for mean(*FD*_*w*_) obtained with IN version were less accurate than with WN, they are not presented. However, std(*FD*_*w*_) obtained with IN version were also considerably smaller than those with Higuchi's or the CD method. As well, because more accurate results were obtained with power than with logarithmic model (lower fitting error on Fig. 4B), these and all following measurements were not performed with the logarithmic model. As expected, when the epoch shortened, std(*FD*_*w*_) increased for all methods (Fig. 5B and 5D). The new NLD method showed a significantly lower *FD*_*w *_scattering than Higuchi's or CD, especially for short epochs, reducing the numerical error. However, this improvement was obtained at the expense of an increased difference between mean *FD*_*w *_and the theoretical *FD *value (Fig. 5, upper panels, **A **and **C**). Fortunately, when extracting complexity waveforms from one-dimensional signals, it is more important to have lower scattering of window *FD *values than the preciseness of their mean value. The latter contributes merely to the "DC" level of the extracted complexity waveform, while the waveform quality (signal-to-noise ratio i.e. window-to-window fluctuations) depends heavily on the standard deviation of the *FD*_*w *_values. This statement will be illustrated on several examples.

### Extraction of complexity waveforms from synthetic signals

**Figure 6:**
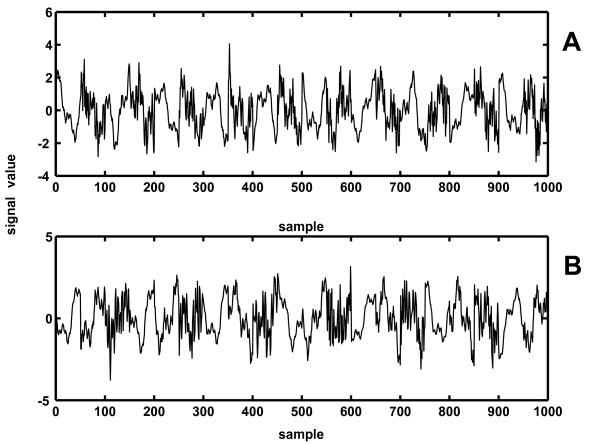
**Two synthetic signals, obtained by alternating 50 sample epochs from two (A) or three (B) Weierstrass functions**. (**A**) *FD*_1 _= 1.2; *FD*_2 _= 1.8; **(B)**: *FD*_1 _= 1.1; *FD*_2 _= 1.5, *FD*_3 _= 1.9.

**Figure 7:**
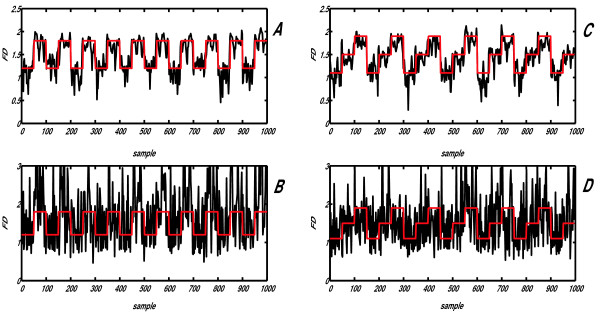
**Results of the comparative analysis, performed by applying the power model IN version of the NLD method (A, C) and Higuchi's method B, D on two synthetic signals, obtained by alternating 50 sample intervals from two Weierstrass functions**. **A, B**: *FD*_1 _= 1.2; *FD*_2 _= 1.8; **C, D**: *FD*_1 _= 1.1; *FD*_2 _= 1.5, *FD*_3 _= 1.9. The two target waveforms (series of rectangular pulses/stairs) are indicated in red.

In order to compare the new method with Higuchi's when dynamical change of signal *FD *occurs, two synthetic signals were constructed from different Weierstrass functions: one consisted of a series of 50 sample epochs, having two alternating *FD *values: 1.2 and 1.8 (Fig. 6A), while the other was formed by composing three *FD *values: 1.1, 1.5 and 1.9 (Fig. 6B). Both signals had γ = 3.4 and were 1000 samples long. This particular value for parameter γ was chosen because its corresponding calibration curve (one of the black lines on Fig. 3) was positioned closest to the average calibration curve (red line on Fig. 3), minimizing the induced systematic error (optimal "DC" level of *FD*(*t*) on Fig. 7A, C). Prior to further processing, each 50 sample epoch underwent amplitude normalization according to expressions (9) and (10), in order to eliminate influence of amplitude differences of componential Weierstrass functions on *FD*(*t*) extraction.

The complexity waveforms of these synthetic signals were analyzed with both power model (WN, IN) versions of the NLD method (Fig. 7A, C and Higuchi's method (Fig. 7B, D), all methods using 5 sample moving epochs, step 2 samples. When compared with the ideal output (series of rectangular impulses/stairs shown in red on all four panels), greater accuracy of the IN version of the new method (WN not shown), presented on panels **A **and **C **of Fig. 7, is obvious. Expressed quantitatively, square error per sample for the first waveform (Fig. 7A, B) was 0.0466 in case of NLD, while 0.5998 for the Higuchi's method (≈12.9 times higher). For the second waveform (Fig. 7C, D), the corresponding figures were 0.0463 and 0.3902 (≈8.4 times higher).

### Final adjustment of the calibration curve based on the analysis of natural (EEG) signals

**Figure 8:**
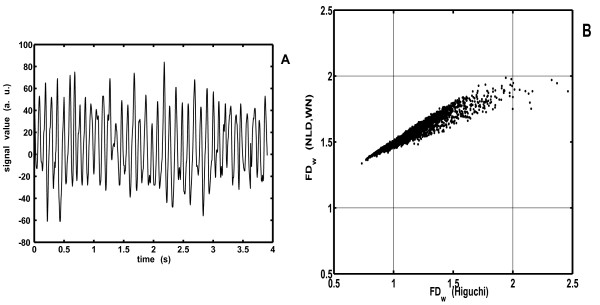
**A. Part of a typical occipital human EEG signal**. **B**. Scatter plot of *FD*_*w *_values, obtained by Higuchi's and power model WN version of the NLD method from the signal presented in **A**, using short windows (10 samples).

The new method and the corresponding calibration curve were constructed by analyzing the formerly described set of Weierstrass functions. However, its applicability on real biomedical signals could only be tested if applied on such natural signals and the obtained results observed critically. We present on Fig. 8 how lower scattering of short window *FD*_*w*_values, obtained with NLD method (compared to Higuchi's), looks on real biomedical signals. In this case a human occipital O1 derivation of the EEG activity was analyzed from a healthy adult in the relaxed awake state with closed eyes.

Fig. 8A presents part of this signal in time domain (4s), while total signal duration was 60s. It was analyzed simultaneously by Higuchi's and the NLD method (power model, WN) with moving non-overlapping short epochs of 10 samples. Resulting values of *FD*_*w *_are given in Fig. 8B in form of a scatter plot. One can observe that points obtained with Higuchi's method are "spilling over" the allowed limits (1<*FD*<2), while in case of the NLD analysis they remain within the marked boundaries. Values calculated with NLD are, however, overestimated in the low *FD *region. We tried to correct this systematic error by constructing a more accurate calibration curve than the one presented on Fig. 4, which was derived only from the Weierstrass functions.

**Figure 9:**
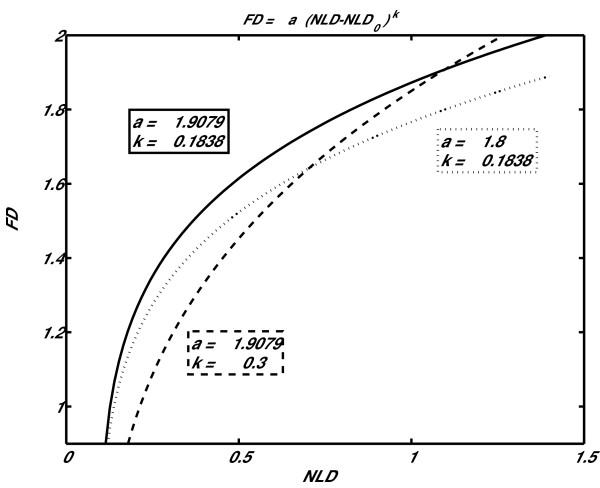
**Influence of power model parameters on the calibration curve**. Initial calibration curve is drawn with solid line. Dotted curve was plotted by decreasing parameter *a *from 1.9079 to 1.8, in case of the dashed curve parameter *k *was increased from 0.18383 to 0.3.

First, we explored how the initial calibration curve is modified when each of the power model parameters (*a*, *NLD*_0 _and *k*) are being varied. In fact, according to the direction and location of the bias shown on Fig 8B, one needs to modify the calibration curve in such a way that *FD *values are decreased in the low *FD *region (left part of the plot), while they stay unchanged in the high *FD *region. Influence of parameters *a *and *k *on the calibration curve are presented on Fig. 9 (parameter *NLD*_0 _was not varied, since it simply shifts the calibration curve horizontally). One can note that increase of *k *(dashed line) decreases *FD *in the low, while decrease of *a *(dotted line) decreases *FD *in the high *FD *region. Therefore, by increasing parameter *k*, the calibration curve is being modified as required – *FD *values decrease in the low, while they remain mostly unchanged in the high *FD *region.

**Figure 10:**
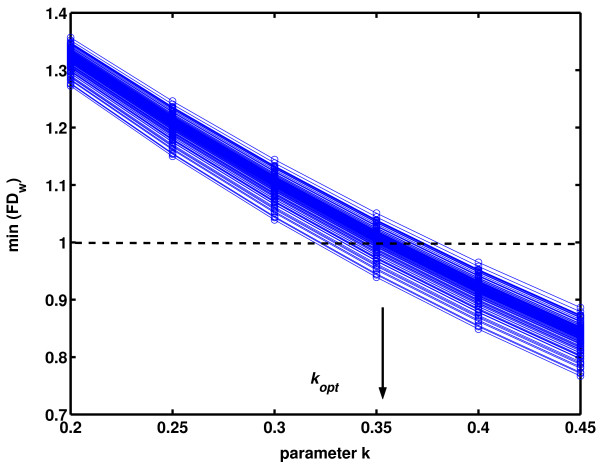
**Minimal *FD*_*w *_values, obtained by NLD method, epoch 10 samples, applied on 140 healthy human EEG signals, as a function of the power model parameter *k***. Each blue line corresponds to one EEG signal. All 140 crossings with min(*FD*_*w*_) = 1 (dashed line) were averaged to calculate *k*_*opt *_= 0.3523.

**Figure 11:**
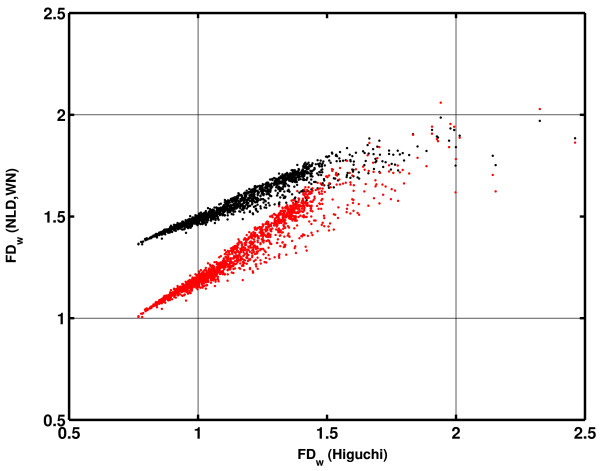
**Scatter plots of 10-sample *FD*_*w *_values of the same human EEG signal as in Fig. 8, obtained by using old (black dots) and the new, corrected (red dots) calibration curve, where the low *FD *region bias is eliminated**.

Further, the optimal new value for *k *could be determined by analyzing a sufficiently large ensemble of biomedical, e.g. EEG signals (so that short epoch *FD*_*w *_values populate more or less evenly all regions of the scatter plot on Fig. 8B) and by observing that all *FD*_*w *_values fall between the required limits (1<*FD*<2). We analyzed 140 EEG signals, each 60s long, from ten adult wake healthy subjects. The electrodes were positioned at 14 locations (F7, F8, T3, T4, T5, T6, F3, F4, C3, C4, P3, P4, O1 and O2) according to the International 10–20 System with an average reference. Signals were sampled at a rate of 256 samples/s, band pass filtered between 0.5 and 70 Hz and artifacts were removed manually based on a visual inspection. Other details about data collection and preparation can be found in [11]. For each signal, a series of 6 analyses (NLD method, epoch 10 samples) were performed, where parameter *k *was varied (0.2 – 0.45, step 0.05). For each of the 140 EEG signals, the minimal *FD*_*w *_was plotted against the varied value of *k *and all 140 crossings with min(*FD*_*w*_) = 1 were determined (Fig. 10). Optimal value for *k *was then calculated as the average of these crossing values: *k*_*opt *_= 0.3523 (± 0.0122). Analogously, as parameter *a *influences the calibration curve in the high FD region, maximal *FD*_*w *_values were also calculated and 140 crossings with max(*FD*_*w*_) = 2 were determined. The obtained optimal value for *a *was *a*_*opt *_= 1.8399 (± 0.0980). Finally, using these new parameters, as an example, we analyzed again the signal from Fig. 8. The corresponding new scatter plot, shown on Fig. 11, confirmed that these corrections (red points) eliminated the formerly detected bias in the low *FD *region.

The method described in this work could find its applicability in those situations where signal complexity changes occur and are of special interest for the researchers. There are two possible cases of such changes:

a) short or intermittent disturbances of the existing signal complexity: external artefacts or internal phasic phenomena, e.g. those occurring in NREM (K-complexes, delta bursts) and in REM sleep (muscle twitches, central and peripheral phasic events such as PGO waves, bursts of autonomic nerves, surges of blood pressure, etc.), as well as microarousal (cf. [12]);

b) slowly evolving FD variations, caused by physiological processes themselves.

In the first case, it is to be expected from the present approach to detect such occurrences, while slower FD variations should be extracted as a function of time (complexity waveforms).

In both cases, it is to be expected that extracted signal complexity changes should be more accurately measured (i.e. less "noisy") if performed by the NLD method than by the existing ones. As well, the new method could be tested in other areas where changes of signal complexity occur, such as engineering, geology, meteorology, astronomy (cf. [13]).

## Conclusion

The new NLD method is more accurate than Higuchi's or consecutive differences for extracting signal complexity waveforms (*FD*(*t*), current changes of signal *FD*), when using short signal epochs (<30 samples). This result follows the fact that standard deviation of its moving window *FD *values (*FD*_*w*_) is considerably smaller than those obtained by the two other methods. However, this improvement is achieved at the expense of lower accuracy in measuring mean *FD*_*w*_. Initially detected overestimation of the *FD*_*w *_values, calculated by NLD in the low *FD *region, was due to an imperfect calibration curve *FD *= f(*NLD*), derived from a set of Weierstrasss functions. The bias was corrected by a procedure of modification of power model parameters, based on the analysis of natural (EEG) signals. The present approach might be used whenever "running" signal *FD *changes are of interest, e.g. in physiological signal analysis for automatic detection of short phasic phenomena or transient artefacts, as well as in other areas where changes of signal complexity occurs, such as engineering, geology, meteorology, astronomy (cf. [12]).

## Competing interests

The authors declare that they have no competing interests.

## Authors' contributions

Idea for the NLD method design was suggested by AK. AK and TB worked on the data analysis. TB and LjR contributed to the interpretation of results. All authors read and approved the final manuscript.
